# Prescribed fire alters nematode communities in an old‐field grassland

**DOI:** 10.1002/ece3.9977

**Published:** 2023-03-31

**Authors:** Min Song, Marshall D. McDaniel, Chen Zhu, Feng Lin, Yaojun Zhang

**Affiliations:** ^1^ International Joint Research Laboratory for Global Change Ecology, School of Life Sciences Henan University Kaifeng Henan 475004 China; ^2^ Department of Agronomy Iowa State University 2517 Agronomy Hall Ames Iowa USA; ^3^ School of Environmental Engineering Nanjing Institute of Technology Nanjing Jiangsu 211167 China

**Keywords:** bottom‐up effect, disturbance, food web, Nematoda, roundworm

## Abstract

Fire is a common disturbance in many biomes, with both beneficial and detrimental effects on soil biology, which largely depend on fire intensity. However, little is known about the impact of fire on soil nematode communities in terrestrial ecosystem. In the present study, we investigated the effects of short‐term prescribed fire on soil nematode communities and soil properties in an old‐field grassland in Northern China. The results showed that burning significantly increased soil nematode abundance by 77% and genus richness by 49% compared to the control. Burning also decreased taxon dominance by 45% (Simpson's *D*) and increased nematode diversity by 31% (Shannon‐Weaver *H*'). However, burning increased plant parasites (particularly genera *Cephalenchus* and *Pratylenchus*) and shifted community to more bacterial‐feeding genera (i.e., decreased Channel Index). Generally, burning increased soil bio‐available nitrogen (NH_4_^+^–N and NO_3_^−^–N) content, which would be the main drivers causing nematode community to flourish via a “bottom‐up” effect. These results suggest that prescribed fire increases nematode diversity and alters community composition toward more plant parasites and bacterial feeders. Our findings highlight the importance of prescribed fire management in shaping short‐term nematode community structure and function, but the long‐term effects and impacts of these changes on soil nutrient and carbon cycling remain unknown.

## Graphical abstract

Prescribed burning increased soil nematode abundance and richness. Prescribed fire significantly altered the nematode community structure. N‐rich food sources would cause the nematode community to flourish via a “bottom‐up” effect.

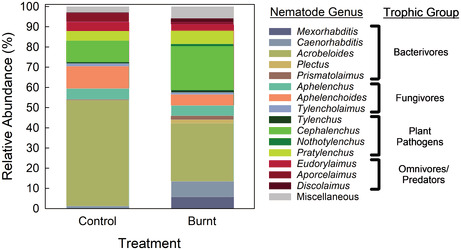

## 1 INTRODUCTION

Fires regulate the structure and function of terrestrial ecosystems, due to their profound effects on vegetation structure and composition, nutrient cycling, and herbivore distribution (Augustine et al., 2014; Bond & Keeley, 2005; Pellegrini et al., 2018; Thoresen et al., 2021). Indeed, some ecosystems are even fire‐dependent and consequently require fire to ensure their successional development (Barnes et al., 2022). At the same time, fire can be used as a landscape management practice, including the annual or semi‐annual burning of grasslands, clearing of debris and encouraging fire‐dependent tree seedling growth, and also used for residue removal in agriculture (Gomes et al., 2020; Thomaz et al., 2014). Given the importance of fire for managing ecosystems and the increasing frequency and intensity of unintentional fires with climate change (Pellegrini et al., 2018), it is critical to understand the consequences of fire for below‐ground ecosystem structure and function.

Most studies that consider the effects of fire on belowground ecosystems mainly focused on soil biogeochemistry and microbes (e.g., bacteria, fungi) generally (Pressler et al., 2019). Soil nematode, however, are critical to soil and general ecosystem function as they prey upon these soil microbes and thereby cycling soil nutrients (Osler & Sommerkorn, 2007; Semeraro et al., 2022). Soil nematode could prevent or cause plant diseases (Wu et al., 2021) and are key predators preying other soil micro‐fauna (Kostenko et al., 2015; Peguero et al., 2019). Neglecting prescribed fire effects on soil nematodes prevents a full picture of the belowground effects of fire on ecosystem structure and functioning (Gomez et al., 2022; Zaitsev et al., 2016).

Nematoda are a widespread and dominant phylum of soil invertebrates, accounting for more than 80% of the multicellular animals in the world (Bloemers et al., 1997). Nematodes colonize nearly all of earth and include a large number of genera with a wide range in size and morphology. They are most commonly known and characterized by their stylet—the specialized organ used for feeding. Nematode communities include almost all trophic groups except for the producer group, and they may feed on bacteria, fungi, or parasitize host plants or animals, according to their feeding habit (Butenko et al., 2017; Moll et al., 2021). Nematodes are also highly sensitive to environmental changes. Nematode community richness and composition respond rapidly to chemical and physical changes (Nielsen et al., 2014). Therefore, nematodes are frequently used as bioindicators of the quality, stability, and changes in the soil (Devi, 2020; du Preez et al., 2018). Therefore, understanding the soil nematodes response to fire is helpful to realize how ecosystem processes are likely to respond to fire.

Previous studies showed inconsistent effects of fire on soil nematode communities. For example, a 4‐year study revealed that fire had strong negative effects on soil nematode community and even reduced soil nematode abundance by 76% in a semiarid grassland (Bastow, 2020). While there are also studies on forest soils affected by fires found that fire significantly increased bacterial‐feeding nematodes abundance even by 640% higher than the unburnt sites (Pen‐Mouratov et al., 2012). Reasons for such inconsistencies are still unclearly, but may be due to environmental (e.g., temperature, pH, N availability) or management differences (e.g., fire intensity) (Matlack, 2001; Trouvé et al., 2020; Zaitsev et al., 2016). In general, this observation is somewhat counterintuitive since many studies demonstrated that soil microbial communities after fires shift from fungi‐dominated to bacteria‐dominated ones (Dooley & Treseder, 2012; Mikita‐Barbato et al., 2015). One can assume that such controversies may be driven by overarching regional differences in their food resource availability and quality, but it is still not known well (Butenko et al., 2017).

In recent decades, major socioeconomic changes in China have led to rural exodus and land abandonment. Agricultural land has been taken out of production and natural regeneration has occurred. As a result, this has produced a series of old‐field successional communities in northern China (Cramer et al., 2008). An inevitable consequence of this successional development has been increased vegetation biomass and hence an added fire risk. Most previous studies of prescribed fire effects on old‐field ecosystems mainly focus on responses of soil physicochemical properties and aboveground ecological processes (Dowhower et al., 2021). How soil nematodes respond to fires in old‐field grassland ecosystems is largely unknown.

To better understand the effects of prescribed fire on soil nematode communities, we conducted an experiment comparing burned and control treatments in an old‐field grassland in Northern China to address the following questions: (1) what are the short‐term effects of fire on the soil nematode abundance and diversity? (2) what are the main factors (e.g., pH, N availability, soil organic carbon (SOC), TN) driving changes in soil nematode communities? and (3) how do functional or trophic groups differ in terms of their response to fire?

## 2 MATERIALS AND METHODS

### 2.1 Study site description

The study was conducted at the Research and Education Farm, Henan University (34°49′16.84′′N, 114°17′56.76′′E, 73 m a.s.l), Kaifeng, Henan. Mean annual precipitation and temperature are 625 mm and 14.4°C, respectively. Soil parent material and texture are Yellow River sediment and sandy loam according to FAO classification, respectively, with 65.7% sand, 14.1% silt, and 20.3% clay (Song et al., 2017). Because these sandy‐loam soils are conducive to greater evaporation combined with the shallow groundwater, slow surface runoff, and high salinity—they are strongly alkaline (pH = 8.7) even though the rainfall is plentiful in this region (Song et al., 2016). Mean annual yield of plant root and aboveground biomass are 210.11 and 330.69 g m^−2^, respectively. The experimental site was originally under wheat‐maize crop rotation until 2000, when the land was left uncultivated and natural successional developed into an “old‐field” grassland. The dominant plant species in the old‐field grassland mainly include *Cynodon dactylon, Gaura parviflora*, *Conyza canadensis, Melilotoides ruthenica*, *Humulus scandens*, and *Setaria viridis*.

### 2.2 Experimental design and sampling

Ten 3 m × 3 m plots were established in the present study. Each plot had 2 m wide buffer zones with removed vegetation and litter to prevent fire spread. Five plots were randomly selected for prescribed burning. For the burning treatment, we fired the withered grass with a blowtorch and burned itself out to mimic the nongrowing season natural fire on December 3, 2018. Soil samples were collected on March 8, 2019. Four cores were randomly taken in each plot with a soil auger (5 cm diameter, 10 cm in depth) and mixed carefully to obtain a total of 10 composite samples. Stones, large roots, and macro‐arthropods were excluded by hand. Each soil sample was divided into three subsamples. The first subsample was used to analyze soil physicochemical properties including soil gravimetric water content, SOC, soil total N (TN), soil pH, NO_3_^−^‐N, and NH_4_^+^‐N. The second subsample was used to measure microbial biomass carbon (MBC) and microbial biomass nitrogen (MBN) concentration. The third subsample was used to extract and identify soil nematodes.

### 2.3 Sample analyses

A total carbon analyzer (Vario MACRO CUBE, Elementar Inc.) was used to analyze soil organic C and total N contents. Soil organic C was determined by the Walkley‐Black's wet digestion method with a Total Organic Carbon Analyzer (Elementar Vario TOC, Elementar Co.). The pH values were determined with a combination glass electrode (soil/water W/V ratio 1:2.5). Microbial biomass was measured by fumigation‐extraction method (Vance et al., 1987). Soil plant‐available, inorganic N—ammonium (NH_4_^+^‐N) and nitrate (NO_3_^−^‐N)—was extracted from 10 g fresh soil with 50 mL 2 M KCl and measured by Discrete Auto Analyzer (SmartChem 200; WestCo Scientific Instruments Inc.).

### 2.4 Soil nematodes extraction procedures and ecological index calculation

Nematodes were extracted from 50 g soil using a modified Baermann method (McSorley & Frederick, 2004). After an extraction of 48 h, nematodes were preserved in 4% formaldehyde, counted, and then adjusted to 100 g dry soil. Subsequently, 100 individuals were identified to genus level using a microscope (400×) and assigned to the following trophic groups: bacterivores (Ba), fungivores (Fu), plant parasites (PP), and omnivores‐predators (Om). Nematode genera were also assigned to colonizer‐persister (c‐p) groups (*cp1‐cp5*) according to Bongers (1999).

Nematode data were also used to calculate Maturity Index (MI) (Yang et al., 2018), Channel Index (CI), Enrichment Index (EI), and Structure Index (SI) (Ferris et al., 2001). Lower and higher values of MI indicate disturbed and stable nematode communities, respectively. CI was calculated to evaluate the relative functional intensity of soil decomposition pathways. A low CI indicates the dominance of the bacterial channel, while a high CI refers the dominance of the fungal channel. A high EI suggests a resource‐enriched soil ecosystem. A high SI indicates a complex and stable food web.

The diversity and ecological indices were calculated as following.

1. Shannon‐Weaver Index: *H*′ = −∑ *p*_*i*_ ln *p*_*i*_, (Yang et al., 2018);
2. Simpson dominance index, *D* = ∑ *p*_*i*_^2^, where *p*_*i*_ is the proportion of individuals in the *i*th taxon (Simpson, 1949);
3. Maturity index: MI = ∑*v*_i_ × *f*_*i*_, where *v*_*i*_ is *c‐p* value of taxon in according to their *r* or *k* characteristics, *f*_*i*_ is the frequency of taxon *i* in the plot (Yang et al., 2018);
4. Chanel index CI = 100 × [0.8 Fu_2_/(3.2 Ba_1_ + 0.8 Fu_2_)] (Ferris et al., 2001);
5. Enrichment index: EI = 100 × ∑ *k*_*e*_ *n*_*e*_/(∑ k_*b*_ *n*_*b*_ + ∑ *k*_*e*_ *n*_*e*_) (Ferris et al., 2001);
6. Structure index: SI = 100 × ∑ *k*_*s*_ *n*_*s*_/(∑ *k*_*b*_ *n*_*b*_ + ∑ *k*_*s*_ *n*_*s*_) (Ferris et al., 2001), where *n*_*b*_ is the abundance of individuals and *k*_*b*_ is the weighting in guilds Ba_1_ and Fu_2_, which represent the basal characteristics of soil food web; *k*_*s*_ is similar weighting assigned to Ba_3_‐Ba_5_, Fu_3_‐Fu_5_, Op_3_‐Op_5_; and *n*_*s*_ is the abundance of above‐mentioned guilds. Ba_*x*_, Fu_*x*_, Op_*x*_ (where *x* = 1–5) represent *c‐p* values and feeding groups following Bongers and Bongers (1998).

### 2.5 Statistical analysis

The nematode population density and genus richness were logarithmically transformed (*Y*′ = ln [*Y* + 1]) and the percentage data (soil moisture, organic matter and relative abundance of soil nematode) were ASIN‐transformed to meet the conditions of normality. All data were analyzed using ANOVAs. Simple linear regressions were used to develop the relationships of nematode population density and genus richness with NO_3_^−^‐N, NH_4_^+^‐N, MBN, MBC, and soil pH. All data analyses were conducted using SAS 9.3 statistical software package (SAS Institute Inc.).

We used principal components analysis (PCA) to determine the prescribed burn effect on the overall nematode community in R (version 3.4.3). Using *prcomp* in *vegan* package (Dixon, 2003; Oksanen et al., 2007), we analyzed for community similarity/dissimilarity. We then used the *envfit* function to correlate ancillary properties (e.g., plant biomass and ancillary soil properties) with nematode community. Finally, a multiple ANOVA, or MANOVA, was conducted using *manova* function with top three principal components from the PCA. Treatment differences at the level of *p* < .05 were considered as significant, but correlates in PCA were mapped with less stringent value of *p* < .1.

## 3 RESULTS

### 3.1 Ancillary soil properties

Three months after the prescribed burn, there was no detectable effect on soil water content, SOC and TN concentration (Table 1). Burning significantly increased NO_3_^−^‐N and NH_4_^+^‐N concentration by 60% and 44%, respectively. Additionally, burning also increased soil MBN and MBC by 44% and 61%, respectively. However, burned sites significantly lowered soil pH (8.76 vs. 8.89).

**TABLE 1:** Soil properties for control and prescribed burn (burnt) treatments (mean ± standard error, *n* = 5)^†^.

Soil property	Units	Control	Burnt
Gravimetric water content	g H_2_O g^−1^	0.14 ± 0.01 a	0.11 ± 0.01 a
Soil organic carbon	g C kg^−1^	39.56 ± 0.95 a	38.65 ± 0.67 a
Total nitrogen	g N kg^−1^	1.54 ± 0.05 a	1.50 ± 0.04 a
Nitrate N	mg N kg^−1^	42.04 ± 3.98 b	67.30 ± 5.59 a
Ammonium N	mg kg^−1^	35.94 ± 2.68 b	51.83 ± 4.06 a
Microbial biomass N	mg N kg^−1^	167.25 ± 22.38 b	269.68 ± 17.13 a
Microbial biomass C	mg C kg^−1^	311.55 ± 23.57 b	548.48 ± 83.61 a
pH	Unitless	8.89 ± 0.02 a	8.76 ± 0.01 b

^†^ Significant difference between means at *p* < .05 shown with lowercase letters.

### 3.2 Nematode abundance and diversity

Across both treatments, we found 29 taxa from 21 nominal families with frequencies of detection ranging from <99% to >1% of the samples (Table S1). Their feeding habits included bacterial feeding (Ba), fungal feeding (Fu), plant parasites (PP), and omnivores‐predators (Om). Nematode taxa at the experimental site represented all the cp (structural guild) categories (Bongers & Bongers, 1998).

**FIGURE 1:**
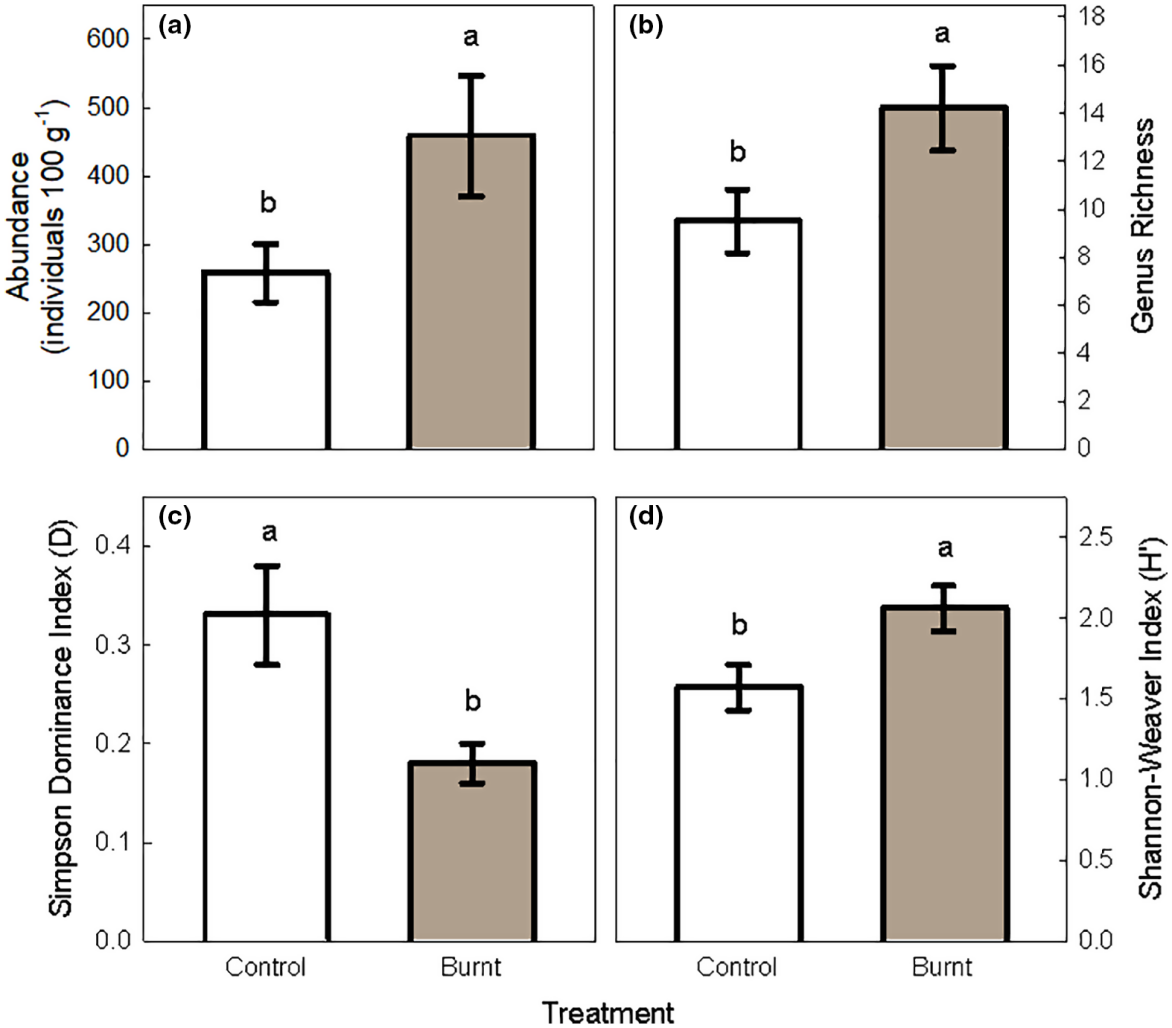
Nematode abundance (a), genus richness (b), Simpson Dominance Index or *D* (c), and Shannon‐Weaver Index or *H* ^′^ (d) for control and prescribed burn (i.e., Burnt). Bars show mean ± standard error (*n* = 5). Lowercase letters indicate significant difference at *p* < .05.

The prescribed burn had strong effects on overall abundance and measures of diversity (Figure 1 and Table 2). Burning increased abundance from 258 ± 43 to 458 ± 88 (mean ± standard error) individual nematodes per 100 g soil, or a 77% increase from the control. Burning also increased genus richness from 9.5 ± 1.3 to 14.2 ± 1.8. Most notable from diversity indices, burning decreased Simpson Dominance Index (*D*) and increased Shannon‐Weaver Index (*H*′) by 45% and 31%, respectively.

**TABLE 2:** Nematode diversity indices description, references, range in values from the literature, and values for control and prescribed burn (burnt) treatments (mean ± standard error, *n* = 5)^†^.

Index	Description and inference	References	Range in values	Control	Burn
Simpson Dominance (*D*)	Simpson's Dominance index can range from 0 to 1; values close to 1 indicate a clear dominance of one or several species	Berbec et al. (2020), Yang et al. (2018)	0.02–0.24	0.33 a	0.18 b
Shannon‐Weaver (*H*′)	The Shannon–Weaver index can be a suitable criterion for the distribution of nematode in soils	Vodyanitskii (2017), Yang et al. (2018)	1.5–1.8	1.57 b	2.06 a
Evenness	These factors are explaining Simpson Dominance (*D*) and Shannon‐Weaver (*H*′)	–	–	0.50 a	0.57 a
Menhinick				1.15 a	1.50 a
Margalef				2.22 a	2.93 a
Equitability_J				0.68 b	0.78 a
Fisher_alpha				3.41 a	4.88 a
Berger‐Parker				0.52 a	0.32 b

^†^ Significant difference between means at *p* < .05 shown with lowercase letters.

### 3.3 Nematode trophic groups and ecological indices

**FIGURE 2:**
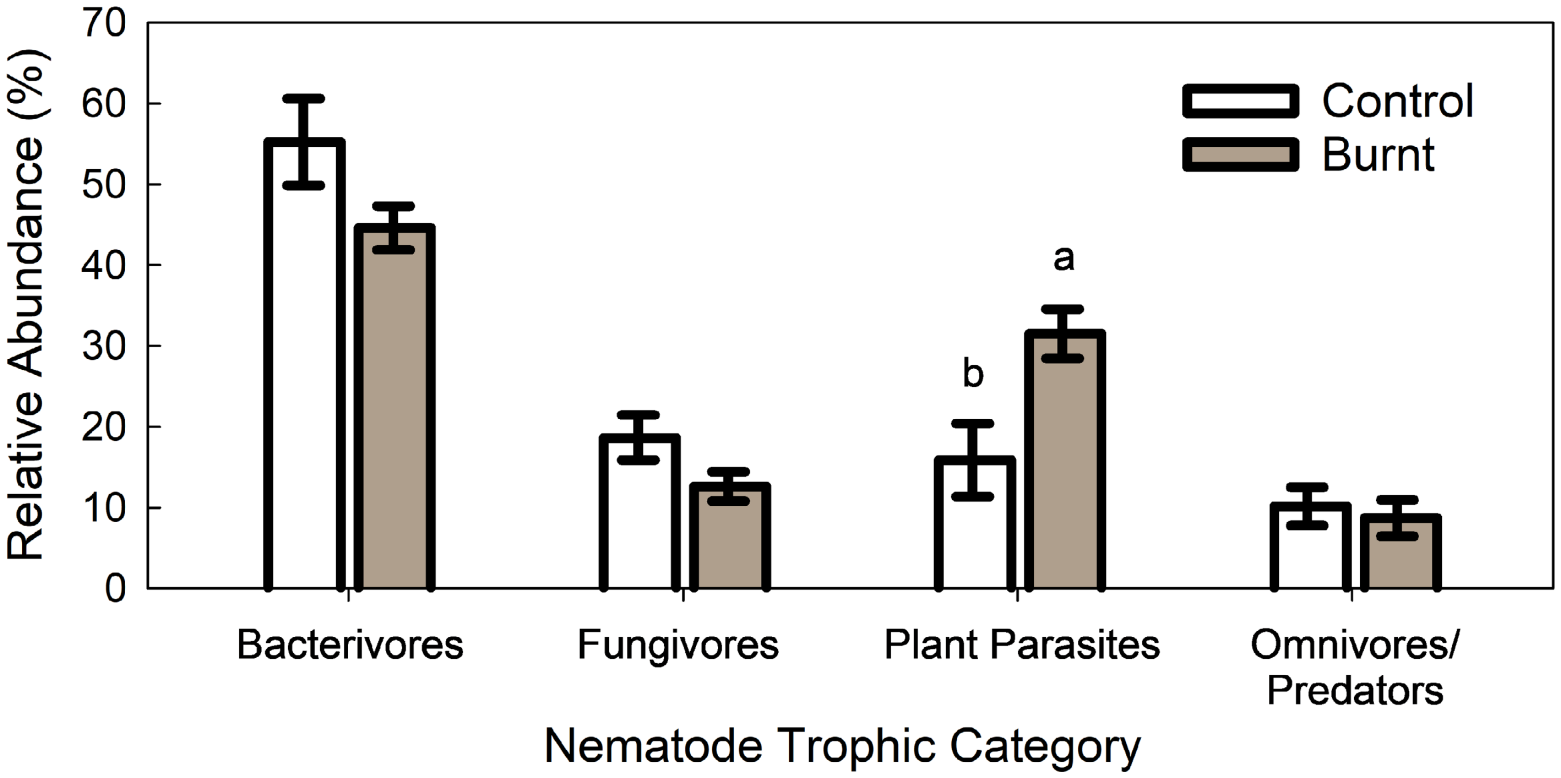
Nematode relative abundance for bacterivores, fungivores, plant parasites, and omnivores/predators for control and prescribed burn (i.e., Burnt). Bars show mean ± standard error (*n* = 5). Lowercase letters indicate significant difference at *p* < .05.

Prescribed burning had striking effects mostly on each trophic group. Burning significantly increased relative abundance of PP by 98% compared to the control (Figure 2).

Prescribed burning had mixed effects on ecological indices (Table 3). Burning increased the EI and the MI, decreased the CI, while had no effect on the SI (Table 3). The EI and CI both point to more resource‐rich environments, since bacteria are considered to proliferate under greater resource availability and after disturbances (i.e., more *r*‐selected), at least compared to fungi.

**TABLE 3:** Nematode ecological indices description, references, range in values from the literature, and values for control and prescribed burn (burnt) treatments (mean ± standard error, *n* = 5)^†^.

Index	Description and inference	Reference	Range in values	Control	Burn
Maturity Index	Proportion of colonizer‐persister genera and indicates disturbed versus stable community	Yang et al. (2018)	1.9–2.6	2.35 ± 0.11 b	3.29 ± 0.06 a
Channel Index	A functional intensity of trophic pathways	Ferris et al. (2001)	10–79	81.10 ± 9.06 a	19.74 ± 7.98 b
Enrichment Index	Resource enrichment of the soil	Ferris et al. (2001)	73–93	23.25 ± 2.24 b	47.63 ± 4.80 a
Structure Index	A measurement of complexity and stability of the nematode community web	Ferris et al. (2001)	4–45	64.84 ± 4.25 a	66.40 ± 2.44 a

^†^ Significant difference between means at *p* < .05 shown with lowercase letters.

### 3.4 Genera composition of nematode community

**FIGURE 3:**
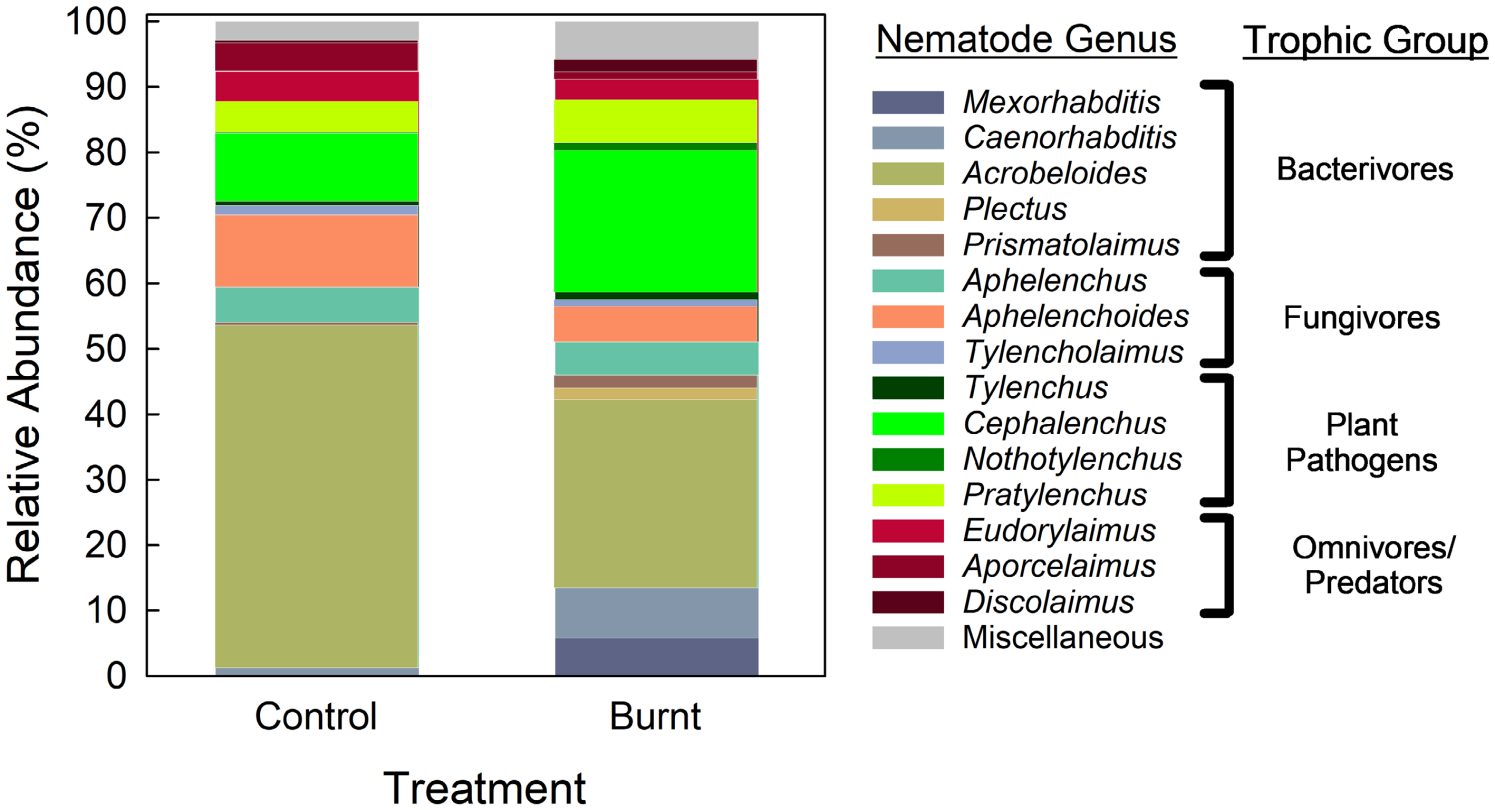
Relative abundance of dominant nematode genera. Dominant genera were deemed those where at least one treatment showed >1% of total abundance. Those with both treatments <1% were grouped into ‘Miscellaneous’ for ease of visualization. Trophic group categories are shown to the right. Abundances are means (*n* = 5).

Prescribed burning had notable effects on nematode genera (Figure 3). Burning decreased the most dominant Ba, *Acrobeloide*, from 49% to 26% relative abundance. Burning also decreased the most abundant Fu, *Aphelenchoides*, from 10% to 4% relative abundance. However, burning increased the dominant PP genus, *Cephalenchus*, from 10% to 22% relative abundance.

**FIGURE 4:**
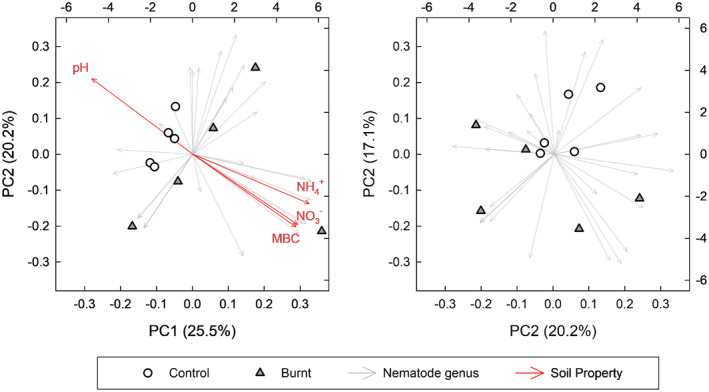
Principal component analysis (PCA) of abundance of nematode genera (individuals per 100 g). Principal components (PC) 1 and 2 shown on LEFT, and PC 2 and 3 shown on RIGHT. The percentage of variance explained for each PC shown in parentheses. Red arrows with labels show correlated ancillary soil properties and environmental variables at (*p* < .1).

By using principal component analysis (PCA), we can both determine overall community differences and elucidate factors driving community shifts. The PCA showed rather low dominance of top principal components (PCs; Figure S1). Plotting PC1 vs. PC2 explained 45.7% of variation and showed distinct separation between control and burned nematode communities. A MANOVA of top three PCs showed that community composition was significantly different (*p* = .036). Soil MBC, nitrate, and ammonium were positively correlated with prescribed burn soil nematode community, but pH negatively correlated (*p* < .1, Figure 4). This can also be confirmed with univariate correlations between these soil properties and both abundance and genus richness (Figure S2).

## 4 DISCUSSION

### 4.1 The underlying effect of fire intensity on soil nematode abundance and diversity

To our surprise, contrary to our hypothesis and prior studies, we found prescribed burning increased soil nematode abundance and richness (Figure 1). Most studies show that fire can decrease soil nematode abundance and richness even by 88% and 96%, respectively (Bastow, 2020; Pressler et al., 2019; Whitford et al., 2014). However, other studies have shown significant positive (+35%) or neutral effects of fire on nematodes (Čerevková et al., 2013; Matlack, 2001). These conflicting responses of soil nematodes to burning probably related to either environmental factors (soils and/or climate) or the intensity of the fire. With fire intensity being the most parsimonious explanation (Whitford et al., 2014). Previous studies have shown that the responses, positive or negative, of soil organic matter (SOM), and microorganism to fire are related to the intensity (Alcañiz et al., 2018). More specifically, low intensity fire can increase soil resource availability and microbial biomass, thus effectively stimulating the activity of soil nematode (Liu et al., 2007; Úbeda et al., 2005). On the contrary, high intensity fire will reduce soil resource availability, and then restrain the supply of nematode nutrition resulting in low soil nematode abundance and richness (Hinojosa et al., 2021; Shakesby et al., 2015). We did not address the fire intensity in our study, but from this we might expect we had lower‐intensity fire with lower maximum temperatures since both nematode abundance and microbial biomass increased with burning (Figure 4).

### 4.2 The main factors driving soil nematode community changes

While fire can have direct effects on soil biota with increased temperatures, there are also indirect effects of fire that many have documented. In our study, prescribed burning altered some environmental variables that have known effects on soil nematode communities. While these relationships do not infer causation, they can help lead hypothesis generation for why nematode communities change with prescribed fires.

Burning rapidly oxidizes the soil organic matter thereby releasing NH_4_^+^‐N (Christensen, 1973; Knoepp & Swank, 1993; Kovacic et al., 1986). Most NH_4_^+^‐N can move downward and condense in the mineral soil as exchangeable N. This NH_4_^+^‐N can also be rapidly converted to soil NO_3_^−^‐N via nitrification (Knoepp & Swank, 1993). The effects of these increased levels of highly available N during and following fire are often beneficial to the recovering plants and microbial populations by providing more bio‐available nutrients (Fultz et al., 2016; Gray & Dighton, 2009; Liu et al., 2007). In our research, the increasing MBN is direct proof for that. As a result, these abundant N‐rich food sources would cause the nematode community to flourish via a “bottom‐up” effect.

The changing soil microenvironment is another important reason for the increase of soil nematode abundance. Soil pH generally increases following fires (Certini, 2005), but decreases have been observed in lower intensity fires (Pereira et al., 2017). Burning decreased soil pH in our study by 1.5% (Table 1). In coarse‐textured sandy soils, the cations released from organic matter during the fire are easily lost from soil due to the surface runoff and erosion, resulting in a decrease in soil pH. In addition, increases in microbial activity (SOM decomposing) and nitrification can also lower pH via proton generation from these biological processes. In order to regulate their osmotic pressure, nematodes exchange several ions through their cuticle (Castro & Thomason, 1971). Changes in soil pH, due to burning, could have led to indirect effects on the nematode community (Korthals et al., 1996; Liang et al., 2020; Räty & Huhta, 2003). In our research, the increase of soil temperature during the burn and the increase of radiation resulting from dark‐colored fuel residues are also important reasons for the increase of soil nematode (Convey & Wynn‐Williams, 2002). In addition, the decreasing soil pH move soil pH closer to neutral would benefit most nematode genera (Burns, 1971; Oka et al., 2000), which means soil pH have bigger indirect impacts on nematodes.

### 4.3 The effect of fire on nematode functional and trophic groups

Prescribed fire significantly altered the nematode community structure (Figures 2 and 3). In particular, burning increased the proportion of PPs by 98% compared to the control (Figure 2). This observation was consistent with previous study showing prescribed fire increased plant pathogen nematodes by 20% in a tall tussock grassland ecosystem after 16 months since last burnt (Yeates & Lee, 1997).

The PP genus with greatest increase was *Cephalenchus*, a widespread genus, its proportion in the burned area was twice as much as that in the control area. In general, plants inhabiting burnt areas are faster growing due to fire‐induced availability of resources, such as light, space, and nutrients (Butenko et al., 2017). In contrast to PP, burning decreased the relative abundance of Fu (and Ba), albeit insignificantly (Figure 2). This could be attributed to the fungi, in particular, are more sensitive to heat and slower to return to pre‐fire levels (Pressler et al., 2019). Previous studies have shown that heating soils can release chemicals that inhibit fungal growth (Choromanska & DeLuca, 2001; Díaz‐Raviña et al., 1996).

Burning increased the Shannon‐Weaver index (*H*') but suppressed the dominance (Figure 1). This was mainly due to the fact that burning increased genus richness but reduced the proportion of dominant genera (e.g., *Acrobeloide* and *Aphelenchoides*). In terms of ecological indices, burning positively affected the EI and the CI, but had no effects on the SI and MI (Table 3). The values of EI could reflect the increased availability of resources to the soil food web and the response of primary decomposers to the resources, which has been found to directly relate to the cumulative net N mineralization (Ferris & Matute, 2003). In this study, burning decreased the CI by 75.7% (Table 2). The changes in CI could be largely explained by the more dramatic decrease in the relative abundance of fungivores than bacterivores. This finding is consistent with meta‐analysis comparison that reported that bacterial community changes were more resistant to fire than fungi across the literature (Pressler et al., 2019), and thus CI has been used as an indicator of bacterial‐vs‐fungal decomposition pathways (Ferris et al., 2001). Low CI values indicate bacterial dominated decomposition whereas high values refer to a more fungal dominated system. In this experiment, the lower CI implies that burning drives the soil food web to bacterial decomposition channels.

## 5 CONCLUSIONS

Fire can have destructive effects on soil nematode communities, as most comprehensively shown in meta‐analysis. However, we found controlled burning had positive effects on nematode abundance and diversity after 3 months. This unique finding could be due to edaphoclimatic context of our study, management factors like intensity of our prescribed burn, or both factors.

While controlled burning increased nematode abundance and diversity, it also increased the relative abundance of plant pathogens. In particular, it increased the abundance of *Cephalenchus*. The long‐term impacts of this disturbance and change in nematode communities are unknown, but it may have short‐term (1–2 years) or even long‐lasting impacts like increasing parasitation of the recovering plant community. Our findings highlight the importance of prescribed fire management in shaping short‐term nematode community structure and function. However, the long‐term effects remain unclear and warrant further exploration, in particular how they might be related to these short‐term changes in nematode community dynamics.

## AUTHOR CONTRIBUTIONS

**Min Song:** Conceptualization (equal); data curation (lead); methodology (equal); writing – original draft (equal); writing – review and editing (equal). **Marshall D. McDaniel:** Data curation (equal); methodology (equal); writing – review and editing (equal). **Chen Zhu:** Data curation (equal); investigation (equal). **Feng Lin:** Writing – review and editing (equal). **Yaojun Zhang:** Investigation (equal); supervision (equal); visualization (equal); writing – review and editing (equal).

## CONFLICT OF INTEREST STATEMENT

The authors declare that they have neither personal relationships nor competing financial interests that could affect the work.

## Supporting information


Data S1

## Data Availability

The data that support the findings of this study are openly available in Dryad at 10.5061/dryad.j3tx95xk9.
