# Supplementary material for: Prescribed fire alters nematode communities in an old‐field grassland
Source: Ecol Evol. 2023 Mar 31;13(4):e9977. doi: 10.1002/ece3.9977 (PMC10065976; doi:10.1002/ece3.9977)
Supplement: Supplementary file 1 — Data S1 [file ECE3-13-e9977-s001.docx]

SUPPLEMENTAL MATERIALS FOR:
**Prescribed fire alters nematode communities and soil properties in an old-field grassland**

Min Song^1^, Marshall D. McDaniel^2^, Chen Zhu^1^, Feng Lin^3^, Yaojun Zhang^1^ *

1 International Joint Research Laboratory for Global Change Ecology, School of Life Sciences, Henan University, Kaifeng, Henan 475004, China

2 Department of Agronomy, Iowa State University, 2517 Agronomy Hall, Ames, IA, United States

3 School of Environmental Engineering, Nanjing Institute of Technology, Nanjing, Jiangsu 211167, China

* Correspondence: Yaojun Zhang, E-mail: [njauyjzhang@163.com](mailto:njauyjzhang@163.com)

Table S1. Abundance of nematodes (individuals 100 g^-1^) for control and prescribed burn (burnt) treatments (mean ± standard error, n = 5) ^†^.

| Family | Genus | Trophic Group | Colonizer-Persister (c-p) Value | Control | Burnt |
| --- | --- | --- | --- | --- | --- |
| Rhabditidae | *Mexorhabditis* | Bacterivore | 1 | 0 | 4.6±2.14 |
| Rhabditidae | *Caenorhabditis* | Bacterivore | 1 | 1±0.45 | 7.2±3.54 |
| Axonolaimidae | *Cylindrolaimus* | Bacterivore | 2 | 0 | 0.2±0.2 |
| Cephalobidae | *Acrobeloides* | Bacterivore | 2 | 48.8±7.39 | 25.6±4.8 |
| Cephalobidae | *Cervidellus* | Bacterivore | 2 | 0.4±0.24 | 0.2±0.2 |
| Cephalobidae | *Arobeles* | Bacterivore | 2 | 0 | 0.8±0.58 |
| Plectidae | *Plectus* | Bacterivore | 2 | 0 | 1.6±1.12 |
| Monhysteridae | *Prismatolaimus* | Bacterivore | 2 | 0.4±0.24 | 1.8±1.2 |
| Alaimidae | *Alaimus* | Bacterivore | 4 | 0.8±0.49 | 0 |
| Aphelenchidae | *Aphelenchus* | Fungivore | 2 | 4.6±1.08 | 4.8±1.68 |
| Aphelenchoididae | *Aphelenchoides* | Fungivore | 2 | 10.4±3.09 | 4.2±1.53 |
| Diphtherophoridae | *Diphtherophora* | Fungivore | 3 | 0 | 0.2±0.2 |
| Tylencholaimidae | *Enchodelun* | Fungivore | 4 | 0.8±0.37 | 0 |
| Tylencolaimellidae | *Tylencholaimellus* | Fungivore | 4 | 0 | 0.8±0.8 |
| Tylencholaimidae | *Tylencholaimus* | Fungivore | 4 | 1.4±1.17 | 1±0.63 |
| Tylenchidae | *Tylenchus* | Plant Pathogen | 2 | 0.6±0.4 | 1±0.77 |
| Tylenchorhynchidae | *Cephalenchus* | Plant Pathogen | 3 | 9.4±3.93 | 20±3.51 |
| Nothotylenchidae | *Nothotylenchus* | Plant Pathogen | 2 | 0.2±0.2 | 1±0.55 |
| Praylenchidae | *Pratylenchus* | Plant Pathogen | 3 | 4.4±1.17 | 6±2.0 |
| Hoplolaimidae | *Helicotylenchus* | Plant Pathogen | 3 | 0 | 0.6±0.4 |
| Tylenchorhynchidae | *Tylenchorhynchus* | Plant Pathogen | 3 | 0 | 0.4±0.4 |
| Dorylaimidae | *Labronema* | Omnivore/Predator | 4 | 0.4±0.4 | 0.6±0.24 |
| Dorylaimidae | *Dorylaimus* | Omnivore/Predator | 4 | 0.2±0.2 | 0.2±0.2 |
| Dorylaimidae | *Eudorylaimus* | Omnivore/Predator | 4 | 3.4±1.57 | 2.8±0.92 |
| Dorylaimidae | *Mesodorylaimus* | Omnivore/Predator | 4 | 0 | 0.8±0.58 |
| Dorylaimidae | *Aporcelaimus* | Omnivore/Predator | 5 | 3.4±1.29 | 1.0±0.55 |
| Dorylaimidae | *Discolaimus* | Omnivore/Predator | 5 | 0.4±0.24 | 1.8±1.11 |
| Belonolaimidae | *Belonolaimidae* | Omnivore/Predator | 5 | 0 | 0.2±0.2 |
| Axonochiidae | *Axonchium* | Omnivore/Predator | 5 | 0.2±0.2 | 0.6±0.4 |

†: Significant difference between means at *p* < 0.05 shown with lowercase letters.


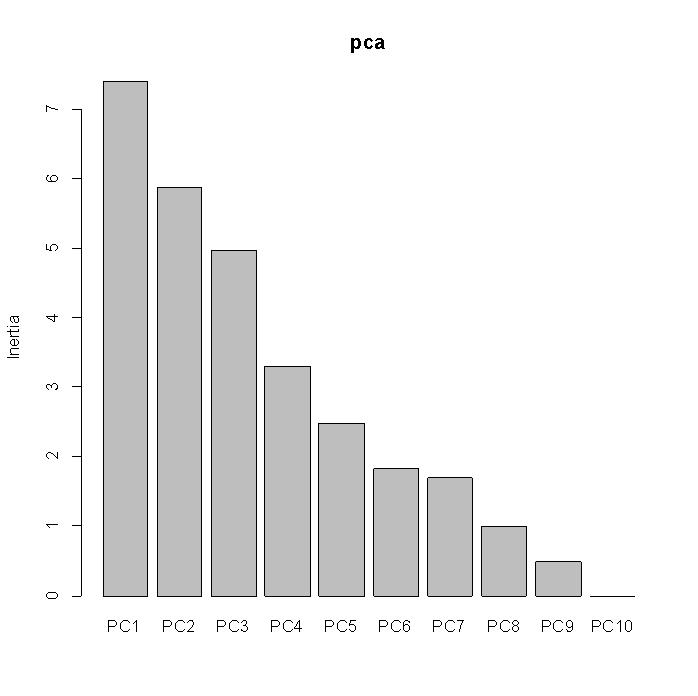


Figure S1. Inertia plot of principal components analysis (PCA) in Figure 5.


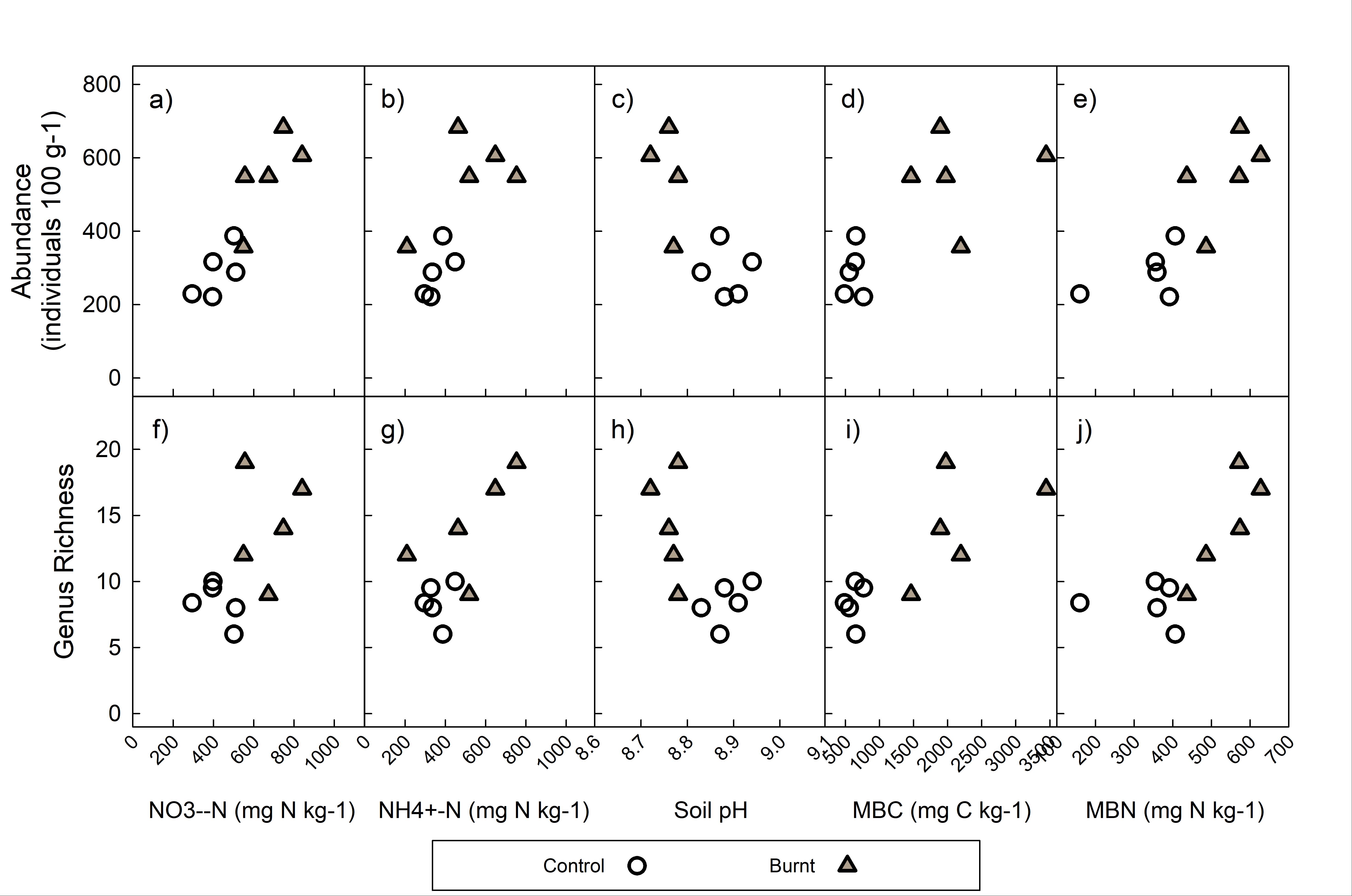


Figure S2. Ancillary soil properties correlated with nematode abundance [a) through e)] and genus richness [f) through j)]. Linear regression line show if significant at *p* < 0.05.
